# In search of work/life balance: trainee perspectives on part-time obstetrics and gynaecology specialist training

**DOI:** 10.1186/1756-0500-5-19

**Published:** 2012-01-10

**Authors:** Amanda Henry, Sarah Clements, Ashley Kingston, Jason Abbott

**Affiliations:** 1 School of Women's and Children's Health, University of New South Wales, Sydney, Australia; 2 Department of Maternal-Fetal Medicine, Royal Hospital for Women, Sydney, Australia

**Keywords:** Education, Medical, Graduate, Personnel staffing and scheduling, Physicians, Women, Inservice training

## Abstract

**Background:**

Part-time training (PTT) is accessed by approximately 10% of Australian obstetrics and gynaecology trainees, a small but increasing minority which reflects the growing demand for improved work/life balance amongst the Australian medical workforce. This survey reports the attitudes and experiences of both full-time and part-time trainees to PTT.

**Methods:**

An email-based anonymous survey was sent to all Australian obstetrics and gynaecology trainees in April 2009, collecting demographic and training status data, data on personal experiences of PTT and/or trainees, and attitudes towards PTT.

**Results:**

105 responses were received (20% response rate). These indicated strong support (90%) from both full-time (FT) and part-time (PT) trainees for the availability of PTT. PT trainees were significantly more likely than FT trainees to be female with children. Improved morale was seen as a particular advantage of PTT; decreased continuity of care as a disadvantage.

**Conclusions:**

Although limited by poor response rate, both PT and FT Australian obstetric trainees were supportive of part-time training. Both groups recognised important advantages and disadvantages of this mode of training. Currently, part-time training is accessed primarily by female trainees with family responsibilities, with many more trainees considering part-time training than the number that access it.

## Background

Part-time trainees represent a small but increasing number of Australian obstetrics and gynaecology (RANZCOG) trainees, from 2% in 2006 to 7% in 2009 [Unpublished data, RANZCOG Training Services Department]. RANZCOG specialty training is a six-year full-time equivalent course, which a trainee usually commences in PGY3 or PGY4. Training includes 4 years of hospital-based training in both obstetrics and gynaecology, and 2 years of Elective training during which time special interest areas may be pursued in a variety of training settings, subject to prospective approval [1]. Assessments are based on both workplace performance and external examination.

Demand among the wider Australian medical workforce for improved work/life balance, including flexible training options, is high among both men and women, and often unmet [2]. Data on training outcomes for part-time training (PTT) regardless of specialty is scant. The data that do exist supports good outcomes for PTT in both the short and long term [3,4]. Arguments against access to PTT include perceived/potential negative impacts on FT trainees and poor patient care [3]. The current study aimed to examine RANZCOG trainee attitudes regarding PTT, including driving factors towards PTT, perceptions by PT trainees of their training, and perceptions of full-time trainees of their part-time colleagues.

## Methods

An anonymous survey was sent by email to all RANZCOG trainees in April 2009, with 3 subsequent reminders. The survey contained sections for all trainees to complete, regardless of training status, and an additional section for those trainees who were currently, or who had previously been, part-time trainees.

Data collected included non-identifying demographic data; current and past training status, full-time (FT) or part-time (PT); whether trainees had ever considered PTT or were considering it in future, including reasons both for considering PTT and why it was decided against if applicable; what trainees (both FT and PT) who had ever worked with PT trainees saw as the major advantages and disadvantages of PTT; whether trainees were broadly supportive of PTT ("Do you support the concept of part-time training?"), and the reasons for their answer. PT trainees were asked questions which evaluated their PT training experience.

The survey was approved for distribution by the Continuing Professional Development Committee of RANZCOG and ethics approval was obtained from the University of NSW. Data were entered into an Excel spread sheet and analysed using SPSS Statistics 17.0 (SPSS Inc, Chicago Ill). Chi-square test and Fisher exact tests were used, as appropriate, to test for significant differences between categorical variables. All statistical tests were 2 sided with statistical significance defined as a probability value of < .05.

## Results and discussion

105 valid responses were received, a response rate of 20% for the total 2009 trainee body: 89 responses (85% of total) were from FT trainees, 15 from PT trainees (11 currently in PTT, 4 previously in PTT), and 1 respondent did not provide their training status.

Demographic characteristics of FT and PT respondents are shown in Table 1. More PT respondents were female and had children than FT respondents. Major stated reasons for PT training were caring for children (67%) and better exam preparation (40%). Of the 9 PT trainees who had sat exams, 7 of 9 passed the written and 7 of 7 the oral specialist Membership exams at their first attempt. Three of 15 PT trainees had not yet sat exams and 3 did not answer this question. PT trainees generally felt that their clinical experience and choice of rotations were equivalent to the corresponding period of FT training, education better or equivalent, research opportunities better or equivalent, but continuity of care worse. Half would have interrupted their training if PT training was not available, and two-thirds intend to undertake PT work at consultant level.

**Table 1:** Demographic characteristics of PT and FT trainees

Demographic characteristics	Part-time trainees (current or previously) - n(%)	Full-time trainees - n(%)
	Total n = 15	Total n = 89
Gender - male	2 (13%)	35 (39%)
- female	13 (87%)*	53 (60%)*

Age range:		
21-30	4 (27%)	31 (35%)
31-40	11 (73%)	45 (51%)
41-50		12 (14%)
51-60		1 (1%)

Marital status:		
Married	10 (67%)	57 (64%)
Defacto	2 (13%)	10 (11%)
Single	3 (20%)	21 (24%)
not stated/unknown		1 (1%)

Children:		
None	3 (20%)#	48 (54%)#
1	5 (33%)	14 (16%)
2	6 (40%)	15 (17%)
3 or more	1 (7%)	11 (12%)
not stated/unknown		1 (1%)

*p = .049 (*χ*2)

#p = .013 (*χ*2)

Of the FT respondents, 26% had previously considered PTT and 44% were considering it in the future. Caring for children (79%) and stress/exams (26%) were major reasons for considering PTT. Extended length of training (91%) and financial considerations (61%) were the major reasons PTT had not been undertaken in this group.

The perceived advantages and disadvantages of working with PT trainees are shown in Table 2. Both FT (27%) and PT respondents (38%) nominated poor continuity of care as the major disadvantage of working with PT trainees, and improved morale and flexible rostering as major advantages. Both FT and PT respondents viewed flexible rostering as an advantage, though more PT respondents viewed it as such.

**Table 2:** Impact on training for trainees who have worked with part-time trainees

	Part-time trainees	Full-time trainees
	n(%) total n = 13	n(%) total n = 62
Advantages of part time training		
- **flexible roster**	10 (78%)*	25 (40%)*
- **improved morale**	6 (46%)	23 (37%)
- care for family member	1 (8%)	17 (27%)
- other		3 (5%)

Disadvantages of part-time training		
- difficulties with rostering	4 (31%)	12 (14%)
- increased workload	1 (8%)	11 (12%)
- **poor continuity of care**	5 (38%)	17 (27%)
- other	2 (15%)	3 (5%)

Do you support the concept of part-time training?	All respondents (n = 105)	By training status
Yes	94 (90%)	FT 80 (90%) PT 14 (93%)
No	6 (6%)	FT 6 (10%) PT 0 (0%)
No response	5 (5%)	

*Significant difference between responses of FT trainee and PT trainee responses (p = .016, *χ*2)

90% of all respondents were supportive of the concept of PTT, with little difference between PT and FT. Most cited work/life balance, improved morale, a general need for flexibility during a long training program, and family commitments as reasons for their support of PTT. The view that the availability of flexible, family-friendly training is important for the specialty training college to be viewed positively by both trainees and the wider Australian community was frequently expressed.

The main limitation of this survey is the disappointing response rate of 20.3%, which is lower than previously published surveys of RANZCOG trainees [5,6]. This was likely partly due to the web-based distribution format using the newly introduced RANZCOG email addresses for all trainees. Technical issues and unfamiliarity with this method of College correspondence may have led to many trainees being unaware of, or not accessing, their RANZCOG emails during the survey period. Given the small total number of PT trainees, it may also reflect that many trainees do not see PTT as an issue of concern. A comparison of gender and age data did not show significant differences between trainees who responded to the survey and those who did not [Unpublished data, RANZCOG Training Services Department], which indicates that the sample may still be representative of the wider trainee body despite the low response rate.

It is anticipated that the distribution and response issues that hampered this survey will be substantially less in future, as use of online course materials during medical school is now almost universal in internet-capable settings [7], and 65% of medical graduates in a recent local survey were sufficiently familiar with social media to have a Facebook account [8]. 'Trainee email' should therefore by the time of follow-up surveys be a well-integrated, routine use of electronic media amongst RANZCOG trainees. To ensure a robust and representative response rate, we would also recommend that future surveys incorporate one paper mail-out, to capture trainees on leave and those who remain infrequent users of information technology.

The findings of the survey were broadly in line with trainee surveys of other specialties and from overseas. The strong support for the concept of PT training (90%) is comparable to that found in an American paediatrics residency of 88% [4] but higher than the 60% found in an Australian paediatric job-share program [9]. Family responsibility was the primary motivator of PTT in this survey, a finding that has been corroborated in other studies [10,11]. Given this motivator, the demography of PT trainees being more likely to be women with children is unsurprising. Similar to one previous report [4], many FT respondents report contemplating PTT, but are deterred by financial issues and increased duration of training. This implies that there is a pool of potential PT trainees who would contemplate PTT were it not for these negative factors.

The advantages of part-time work (improved morale, flexible roster) were identified more often than the disadvantages (poor continuity of care, difficulty with rosters, increased workload) by both PT and FT trainees. While PT respondents nominated certain advantages of PT training (flexible rostering) more often than FT trainees, they also nominated potential disadvantages more often, which would suggest that a more balanced view is held by these trainees. It is also encouraging that when compared with their American paediatric colleagues, Australian obstetric FT trainee respondents were less likely to nominate increased workload (12% vs. 43%) and difficulties with rostering (14% vs. 52%) as issues [4].

Both access to and the outcomes from PT training are passionately debated from a scant evidence base. Partly the uncertainty regarding outcome is due to small numbers of PT trainees - even in the UK, where PT training has been available for many years, only 11% of specialist obstetric trainees work PT [12], and a US survey of a residency program that supports PT options had only 24 PT trainees over a 10 year period [4]. In a UK cohort, 120 PT trainees rated their clinical experience equivalent to, and educational experience better than, FT trainees [13], as did our PT obstetrics and gynaecology trainees. In the longer term, UK follow-up of PT trainees from 1972-93 found most finished training by age 40 and 73% held consultant or academic posts [3], while USA specialty board scores have been found to be equivalent amongst FT and PT paediatric trainees [4]. There are no identifiable reports in the literature to substantiate inferior outcomes for PT training. In this survey, there was no self-reported evidence of poor exam outcomes for PT respondents, although the small sample size is acknowledged. Priorities for further study include correlation of training status with outcomes of training and long-term career paths.

## Conclusions

Although limited by poor response rate, the survey found strong support amongst both FT and PT Australian obstetrics and gynaecology trainees for PTT. This training is currently mostly accessed by women with children due to family responsibilities, but over 50% of trainees contemplate PTT, and this unmet need for flexible training is an important issue for postgraduate medical training programs to acknowledge. Although both FT and PT trainees recognise potential clinical difficulties with PTT, these were outweighed by advantages such as improved morale and flexible rostering.

## Abbreviations

FT: Full-time; PGY: Post-graduate year; PT: Part-time; PTT: Part-time training; RANZCOG: Royal Australian and New Zealand College of Obstetricians and Gynaecologists; UK: United Kingdom; USA: United States of America.

## Competing interests

At the time of the survey distribution, AH was the Chair of the Trainees' Committee of the RANZCOG. JA is the Chair of the NSW Training Accreditation Committee of the RANZCOG. This was an unfunded study. The survey was distributed electronically and free of charge by the Training Services Department of the Royal Australian and New Zealand College of Obstetricians and Gynaecologists to registered RANZCOG trainees of the year 2009.

## Authors' contributions

AH revised the original survey, obtained approval for survey distribution from the RANZCOG, obtained ethics approval, analysed the survey data, and was principally responsible for writing the manuscript. SC created the original survey and performed the literature search. AK converted the survey into a form suitable for electronic distribution, and created and maintained the survey database. JA provided the original study concept, and participated in creation and revision of the survey. All authors read and approved the final manuscript.
